# Preface to Special Topic: Transactions from the 66th Annual Meeting of the American Crystallographic Association

**DOI:** 10.1063/1.4986292

**Published:** 2017-06-14

**Authors:** Arwen R. Pearson, Jason B. Benedict

**Affiliations:** 1 CFEL, Luruper Chausse 149, Universität Hamburg, Hamburg 22761, Germany; 2 771 Natural Sciences Complex, University at Buffalo, Buffalo, New York 14260-3000, USA

Spanning time scales from seconds to femtoseconds, the rapidly growing field of structural dynamics explores both equilibrium and non-equilibrium processes in chemical, biological, and condensed matter systems. Advances in X-ray sources and detectors as well as new approaches for sample delivery, data collection, and processing continue to drive exciting discoveries in atomic and electronic motion in systems large and small.

In this special issue of Structural Dynamics, issue 3 of 2017, the research groups contributing these seven manuscripts are representative of the rich diversity of research that was presented during the Transactions Symposium of the 66th Annual Meeting of the ACA in Denver, CO. The two review articles nicely summarize the evolution of structure-based research involving Photoactive Yellow Protein and the critical role of microfluidics in the crystallization and structure determination of proteins. The invited research manuscripts provide key insights into new data processing methodology, small molecule photocrystallography, protein mutagenesis, and membrane protein transport dynamics.

The special issue contributions are summarized as follows:

• Schmidt: Brief review of photoinduced structural dynamics of photoactive yellow protein.
• Pearce and co-workers: Pan-Dataset Density Analysis method improves the reliability and versatility of crystallographic fragment screening for investigating protein-ligand interactions.
• Carter, Jr., and co-workers: Multi-mutant and modular thermodynamic cycles reveal energetic pathways for amino acid activation in Tryptophanyl-tRNA synthetase.
• Coppens: Historical perspective of the birth and evolution of X-ray photocrystallography.
• Chandrasekaran and Carter: Improvements to the PATH algorithm for identifying conformational transition states in functional proteins.
• Shah and co-workers: Molecular dynamics simulations combined with X-ray diffraction experiments reveal key details of hydrolysis and Na^+^/H^+^ pumping in membrane-integral proteins.
• Sui and Perry: Review of microfluidic technologies applied to protein crystal growth and sample delivery in serial crystallography experiments.

On behalf of all of the authors who contributed to the Transaction Symposium and this special issue of Structural Dynamics, we thank you for reading and hope that you find these papers informative and beneficial for your own research.

